# 显著出凝血异常起病的t（8;16）（p11;p13）急性髓系白血病1例报告并文献复习

**DOI:** 10.3760/cma.j.cn121090-20241130-00503

**Published:** 2024-12

**Authors:** 佳雯 戴, 峰 薛, 葳 刘, 磊 张, 仁池 杨, 晓帆 刘

**Affiliations:** 1 中国医学科学院血液病医院（中国医学科学院血液学研究所），血液与健康全国重点实验室，国家血液系统疾病临床医学研究中心，细胞生态海河实验室，天津市血液病基因治疗研究重点实验室，中国医学科学院血液病基因治疗重点实验室，天津 300020 State Key Laboratory of Experimental Hematology, National Clinical Research Center for Blood Diseases, Haihe Laboratory of Cell Ecosystem, Tianjin Key Laboratory of Gene Therapy for Blood Diseases, CAMS Key Laboratory of Gene Therapy for Blood Diseases, Institute of Hematology & Blood Diseases Hospital, Chinese Academy of Medical Sciences & Peking Union Medical College, Tianjin 300020, China; 2 天津医学健康研究院，天津 301600 Tianjin Institutes of Health Science, Tianjin 301600, China; 3 中国医学科学院北京协和医学院群医学及公共卫生学院，北京 100730 School of Population Medicine and Public Health, Chinese Academy of Medical Sciences and Peking Union Medical College, Beijing 100730, China

## Abstract

伴t（8;16）（p11;p13）急性髓系白血病是一类罕见的急性髓系白血病（AML）亚型。本文回顾性分析1例伴t（8;16）（p11;p13）AML患者的临床特点及诊治过程，并进行相关文献复习。患者，女，19岁，因“皮肤瘀斑、消化道出血”入院，迅速进展至弥散性血管内凝血。骨髓形态学模拟异常早幼粒细胞，临床首诊为急性早幼粒细胞白血病。染色体分析发现t（8;16）（p11.2;p13.3）异常，确诊为急性单核细胞白血病。经强化诱导化疗后达完全缓解，巩固化疗维持期间复发，总体预后不良。伴t（8;16）（p11;p13）AML相对罕见，具有特殊的临床表现和实验室特点，预后不良，临床医师应早期识别。

## Abstract (en)

Acute Myeloid Leukemia with t（8;16）（p11;p13）is a rare subtype of AML. This article presents a retrospective analysis of a 19-year-old female patient with t（8;16）（p11;p13）AML, focusing on her clinical features and treatment course, alongside a review of relevant literature. The patient was admitted due to skin ecchymosis and gastrointestinal bleeding, rapidly progressing to disseminated intravascular coagulation. Bone marrow examination revealed an abnormal blast morphology resembling early promyeloblasts, and the initial diagnosis was acute promyelocytic leukemia. However, chromosomal analysis identified the t（8;16）（p11.2;p13.3）translocation. The patient was finally diagnosed with acute monocytic leukemia. Following intensive induction chemotherapy, she achieved complete remission, but relapse occurred during consolidation therapy. The overall prognosis was poor. AML with t（8;16）（p11;p13）is relatively rare and characterized by distinct clinical and laboratory features, with a generally unfavorable prognosis. Early recognition by clinicians is crucial for appropriate management.

伴t（8;16）（p11;p13）染色体异常的急性髓系白血病（AML）是一种罕见病的AML亚型，在AML中发病率不足1％[1]。伴有此类遗传性异常的AML预后不良，2022年欧洲白血病网（ELN）在成人AML诊断和管理指南中将其纳入预后不良危险分层[2]。t（8;16）（p11;p13）AML作为一类特殊的AML亚型，具有特殊的临床表现和实验室检查[3]。由于此类AML发病率极低，国内鲜有报道，临床医师对该病的认识不足。现报告中国医学科学院血液病医院诊治的1例伴t（8;16）（p11;p13）的白血病患者，并进行相关文献复习。

## 病例资料

患者，女性，19岁，2019年2月因皮肤瘀斑20 d，鲜血便3 d就诊我院。外院查凝血功能示凝血酶原时间（PT）19.2 s，活化部分凝血活酶时间（APTT）43.1 s。我院急诊予维生素K_1_经验性治疗2 d，效果欠佳，遂于2019年2月28日收入我科。入院阳性体征：躯干、四肢可见大片瘀斑。辅助检查：凝血功能：PT 19.3 s（正常参考值：10～14 s），APTT 32.2 s（正常参考值：23.7～36 s），凝血酶时间（TT）25.5 s（正常参考值：13.3～19.3 s），D-二聚体4.33 mg/L FEU（正常参考值：0～0.55 mg/L FEU），纤维蛋白原分解产物（FDP）42.5 µg/ml（正常参考值：<0.5 µg/ml），血浆纤溶酶原活性64.5％（正常参考值：75％～150％），血浆α2纤溶酶抑制物活性33.1％（正常参考值：80％～120％）；凝血因子：FⅨ 137.9％（正常参考值：50％～120％），FⅩ 47.8％（正常参考值：47.8％），FⅪ 163.3％（正常参考值：50％～120％）, 蛋白C活性55.5％（正常参考值：70％～140％），蛋白S活性42.2％（正常参考值：70％～123％）；抗磷脂抗体：抗心磷脂抗体、抗β2糖蛋白1抗体阴性，DRVVT筛选63.6 s（正常参考值：31～44 s），DRVVT确认44.1 s（正常参考值：30～38 s）；肝肾功能：LDH 1770.4 U/L（正常参考值：0～248 U/L），余正常；抗核抗体+ENA抗体谱阴性。头胸腹CT未见明显异常。

患者入院后凝血功能进一步恶化，纤维蛋白原降至0.2 g/L，予人纤维蛋白原3 g静脉输注支持治疗。血常规回报WBC 10.37×10^9^/L，HGB 101 g/L，PLT 97×10^9^/L；外周血白细胞分类：幼稚细胞占46％。完善骨髓穿刺+病理活检，骨髓涂片镜下可见原始细胞胞质内大量嗜天青颗粒及核分裂（图1）。结合患者显著出凝血异常表现，考虑急性早幼粒细胞白血病（APL）不除外，遂予全反式维甲酸诱导分化、羟基脲降白细胞，同时继续输注纤维蛋白原、新鲜冰冻血浆支持治疗。患者外周血三系进行性恶化，凝血功能未见改善，迅速进展至显性弥散性血管内凝血（DIC）。诱导分化治疗第4天，骨髓相关结果回报，骨髓细胞形态学：增生极度活跃，幼稚单核细胞占55％，特异性酯酶（CE）阳性率36％，过氧化酶染色（MPO）阳性率98％，PAS染色阳性率100％（细颗粒弥散状）。流式细胞术：异常细胞群占有核细胞43.9％，强表达HLA-DR、CD33、CD64、CD13,表达CD38、MPO,部分表达CD11b、CD14、CD36、CD71、CD56,弱表达CD15、CD4、CD19,不表达CD34、CD117、CD16、CD7、cCD3、cCD79a、TdT、CD10。染色体核型：46,XX,t（8;16）（p11.2;p13.3）[8]/46,XX[12]。白血病43种常见融合基因筛查：阴性。结合上述检查结果，患者确诊急性单核细胞白血病（AML-M_5_），遂调整化疗方案为HIA诱导化疗（高三尖杉酯碱3 mg第1～6天；阿糖胞苷200 mg 第1、3、5天，100 mg第2、4、6天；伊达比星20 mg第1、3天，10 mg第2天）。治疗后患者肿瘤负荷下降，凝血功能逐步改善。2019年4月复查骨髓，原始细胞偶见（图2），微小残留病（MRD）转阴，临床达到完全缓解，后转入白血病科继续规律化疗。

**图1:**
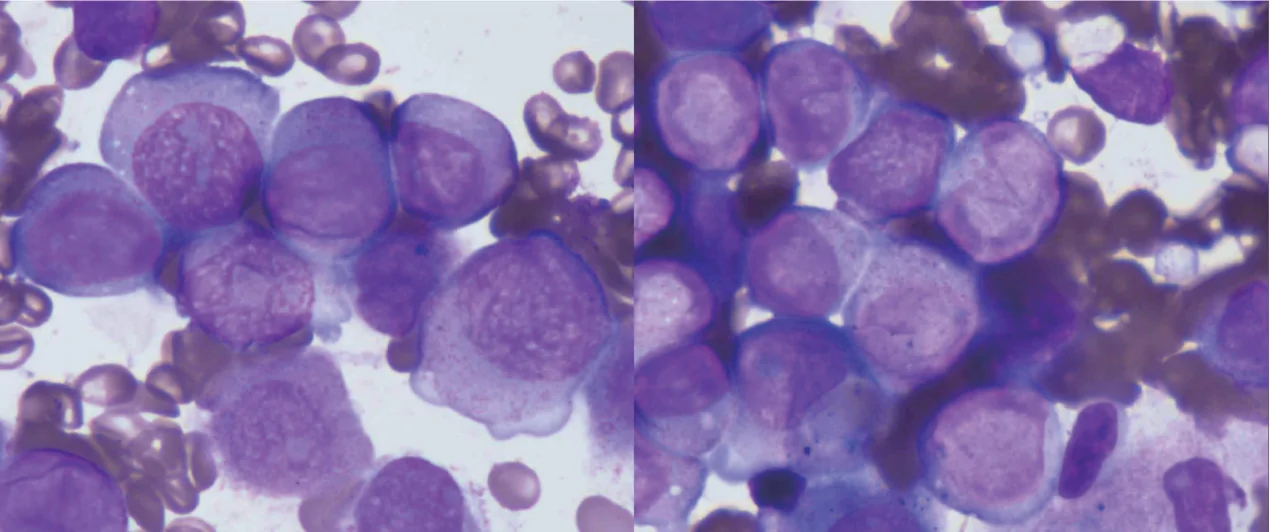
患者治疗前骨髓涂片瑞氏-吉姆萨染色

**图2:**
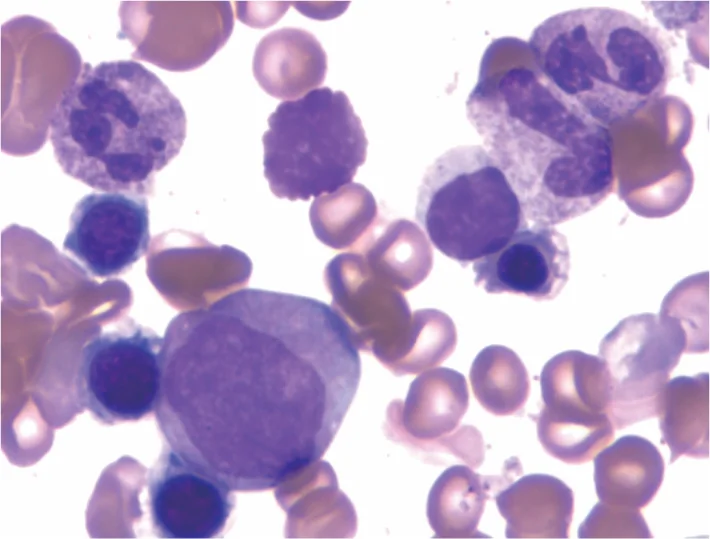
患者治疗后骨髓涂片瑞氏-吉姆萨染色

2020年5月2日患者因“皮肤瘀点”再次就诊我院急诊，查血常规：WBC 5.49×10^9^/L，HGB 121 g/L，PLT 58×10^9^/L；凝血功能：FDP 28.5 ug/ml，纤维蛋白原0.78 g/L，D-二聚体12.49 mg/L FEU，急诊予输注纤维蛋白原支持治疗。患者症状未好转，迅速出现多处骨痛、头痛、呕吐，再次收入我科。入院后患者凝血功能进一步恶化，复查骨髓涂片见异常原始及幼稚单核细胞约占33.5％。诊断AML-M_5_复发，合并DIC，再次予HIA方案诱导化疗。但治疗过程中患者凝血功能进一步恶化，伴多脏器功能衰竭，无法耐受化疗，后患者家属选择自动离院。

## 讨论及文献复习

伴t（8;16）（p11;p13）AML是一类罕见的AML亚型，在AML中占比不足1％[1]。致病机制主要为8号染色体上的MYST3基因与16号染色体上的CREBBP基因发生断裂、重排，继而导致发病[4]–[5]。

1987年，Heim等[6]报道了3例t（8;16）AML-M_5_患者，包括1例10月龄男性患儿和2例女性（15、17岁）患者。3例患者均存在髓外侵犯，其中2例患者骨髓中观察到活跃的吞噬红细胞现象，2例患者诊断后短期内死亡。因此，Heim等提出，伴t（8;16）AML可能是AML-M_5_中的一类特殊亚型。之后陆续有个案报道，任何年龄均可发病，大部分患者存在DIC或者原发纤溶亢进[7]–[11]。1993年Quesnel等[12]报道了6例治疗相关t（8;16）AML，均为肿瘤化疗±放疗后继发AML，5例患者接受了烷化剂治疗，4例患者接受了蒽环类药物治疗，从第一肿瘤到诊断白血病的中位时间为9个月。2013年Diab等[13]报道了18例伴t（8;16）AML，大部分患者存在凝血异常，其中8例患者因骨髓和外周血中早幼粒细胞增多且原始细胞均有APL细胞典型的核分裂而被首诊为APL。近年来国内也偶有t（8;16）AML的个案报道[14]–[16]。

伴t（8;16）AML总体预后不佳，中位生存时间6.7～8.5个月，5年生存率为17％。其中，治疗相关t（8;16）AML的预后明显差于原发AML（5年生存率0％对28％），大部分患者在诊断1个月内死亡[3],[13],[17]。治疗方面，虽然强化诱导化疗后84％的患者获得完全缓解（CR），但大部分患者最终复发。达到CR后接受自体造血干细胞移植的患者5年生存率高于接受巩固化疗的患者（38％对11％）。2022年欧洲白血病网成人AML诊断和管理指南中将t（8;16）（p11;p13）AML正式定义为一个新的白血病亚型，并纳入预后不良危险分层组[2]。2023年成人AML中国诊疗指南也将此类白血病纳入预后不良危险分层组[18]。

本例患者以皮肤瘀点起病，以迅速进展的凝血异常为主要临床表现，骨髓涂片原始细胞胞内嗜天青颗粒丰富伴核分裂，形态学模拟异常早幼粒细胞，因此临床首诊为APL。后根据MICM结果修正诊断为AML-M_5_，经HIA方案诱导化疗后达CR，后继续接受巩固化疗。15个月后患者疾病复发，病情进展迅速，总体预后不佳。

综上所述，伴t（8;16）（p11;p13）AML是AML的一种罕见的特殊亚型，任何年龄均可发病，部分与既往接受放/化疗相关。临床上常表现为显著的凝血异常，伴有高频的髓外侵犯。大部分患者骨髓可见吞噬红细胞现象，部分患者骨髓肿瘤细胞类似异常早幼粒细胞，导致临床误诊为APL，与APL鉴别诊断主要依赖细胞遗传学检测。目前尚无有效的治疗措施，早期确诊、骨髓移植和针对基因的靶向治疗或可提高此类患者的生存率。
